# Biological Equivalence of GGTA-1 Glycosyltransferase Knockout and Standard Porcine Pericardial Tissue Using 90-Day Mitral Valve Implantation in Adolescent Sheep

**DOI:** 10.1007/s13239-021-00585-0

**Published:** 2021-11-24

**Authors:** Christopher McGregor, Jacob Salmonsmith, Gaetano Burriesci, Guerard Byrne

**Affiliations:** 1 Institute of Cardiovascular Science, University College London, London, UK; 2 Department of Surgery, University of Minnesota, 8195B, MMC 195 Mayo, Minneapolis, MN 55455 USA; 3 Department of Mechanical Engineering, University College London, London, UK; 4 Ri.MED Foundation, Bioengineering Group, Palermo, Italy

**Keywords:** Biological heart valve, Xenogeneic antigens, Gal knockout, Tissue equivalency

## Abstract

**Objective:**

There is growing interest in the application of genetically engineered reduced antigenicity animal tissue for manufacture of bioprosthetic heart valves (BHVs) to reduce antibody induced tissue calcification and accelerated structural valve degeneration (SVD). This study tested biological equivalence of valves made from Gal-knockout (GalKO) and standard porcine pericardium after 90-day mitral valve implantation in sheep.

**Methods:**

GalKO (*n* = 5) and standard (*n* = 5) porcine pericardial BHVs were implanted in a randomized and blind fashion into sheep for 90-days. Valve haemodynamic function was measured at 30-day intervals. After explantation, valves were examined for pannus, vegetation, inflammation, thrombus, and tissue calcification.

**Results:**

Nine of 10 recipients completed the study. There was no difference between study groups for haemodynamic performance and no adverse valve-related events. Explanted BHVs showed mild pannus integration and minimal thrombus, with no difference between the groups. Limited focal mineral deposits were detected by x-ray. Atomic spectroscopy analysis detected tissue calcium levels of 1.0 µg/mg ± 0.2 for GalKO BHVs and 1.9 µg/mg ± 0.9 for standard tissue BHVs (*p* = 0.4), considered to be both low and equivalent.

**Conclusions:**

This is the first demonstration of biological equivalence between GalKO and standard pig pericardium. The GalKO mutation causes neither intrinsic detrimental biological nor functional impact on BHV performance. Commercial adaptation of GalKO tissue for surgical or transcatheter BHVs would remove the clinical disparity between patients producing anti-Gal antibody and BHVs containing the Gal antigen. GalKO BHVs may reduce accelerated tissue calcification and SVD, enhancing patient choices, especially for younger patients.

**Graphical Abstract:**

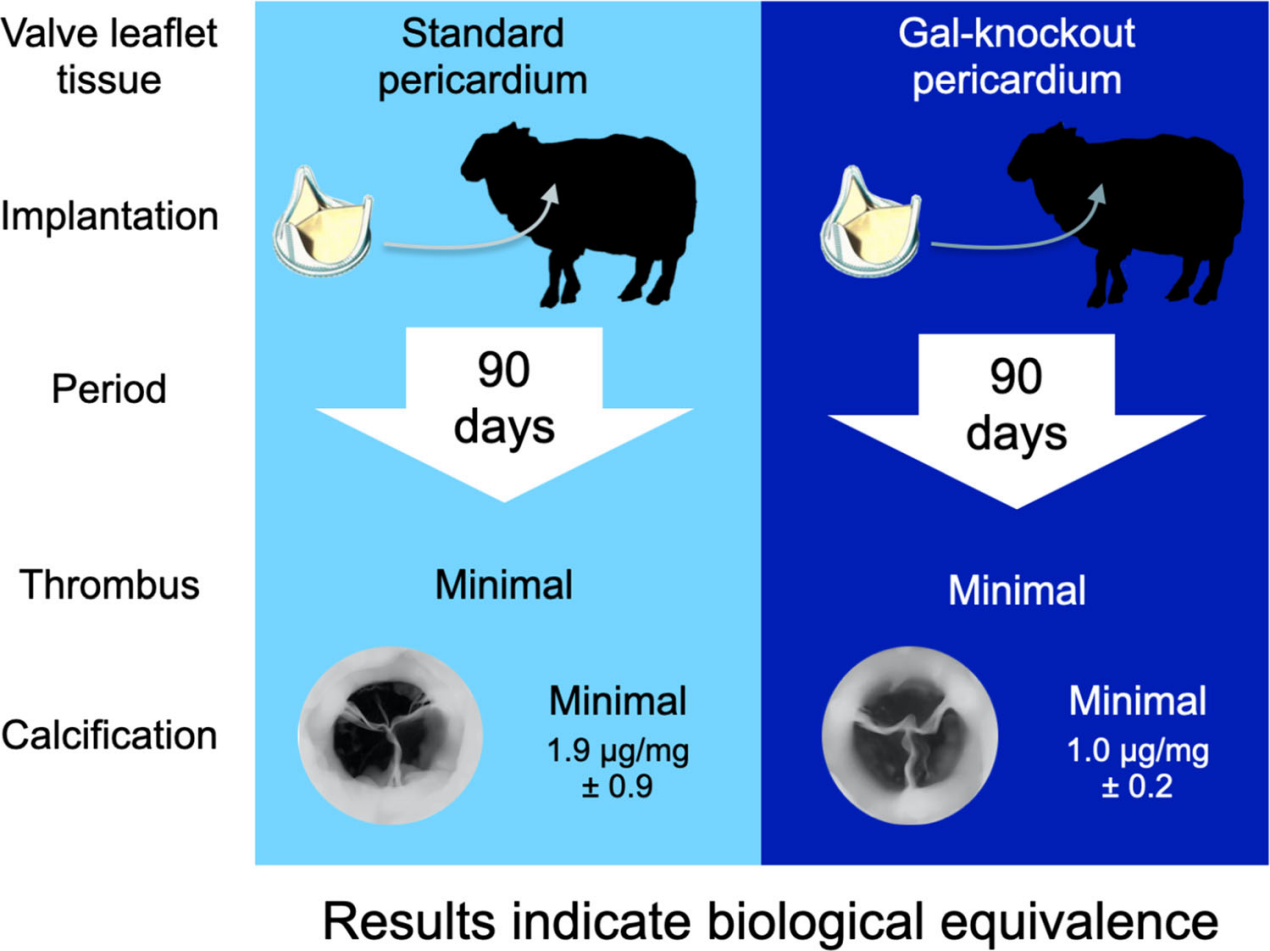

**Supplementary Information:**

The online version contains supplementary material available at 10.1007/s13239-021-00585-0.

## Introduction

Replacement heart valves are highly effective and have been used to treat heart valve diseases for over 50 years.^[6,13]^ Patients with heart valve disease choose between biological heart valves (BHVs), which do not generally require long-term anticoagulation therapy but have more limited durability, and mechanical heart valves (MHVs), which are highly durable but require lifetime anticoagulation, exposing the patient to chronic hemorrhagic/thromboembolic risks.^[16]^ BHV durability is limited by structural valve degeneration (SVD), characterized by leaflet calcification, fibrosis, inflammation, stenosis and leaflet tearing.^[31]^ The occurrence of SVD is strongly age-dependent and, as a consequence, the use of BHVs has largely been abandoned in children and teenagers, as reoperation due to SVD is required in over 90% of cases within 5 years.^[28,31]^ The real-world practice of prosthetic heart valve implantation into adults continues to evolve, and recent AHA/ACC recommendations state that BHVs should be used for patients over 70 years of age and the choice between MHV and BHV implantation individualized for each patient aged 50-70 years.^[28]^ Despite the concerns with re-operation due to SVD, BHVs are popular with patients and physicians, and their use in the U.S. has increased greatly over the last 20 years.^[17]^

To prevent tissue calcification and SVD, various post-glutaraldehyde-fixation chemical treatments have been developed. These treatments commonly extract calcium-binding phospholipids from the fixed tissue and have been highly effective in reducing tissue calcification in preclinical animal models.^[8,9,14]^ There is, however, no clear clinical evidence that these techniques prevent age-dependent SVD or significantly improve BHV durability in patients.^[1,13,28]^ This suggests that another mechanism, independent of passive calcium binding to residual phospholipids, remains a critical clinical barrier to improving BHV durability.

Glutaraldehyde-fixation masks a wide range of protein antigens present in bioprosthetic animal tissue but crucially does not mask the dominant xenogeneic glycan, galactose-alpha-1,3-galactose (Gal), which remains present in high levels in porcine and bovine BHV tissue and clinical devices. Humans and Old World primates do not produce the Gal antigen, but do produce abundant levels of anti-Gal antibody.^[12]^ This creates a clinical disparity between patients producing anti-Gal antibody and BHVs containing the Gal antigen. Clinically, a BHV-specific induction of anti-Gal antibody has been reported in recipients,^[18,21]^ with a stronger response reported in children,^[29]^ showing that the Gal antigen remains immunogenic. We confirmed this immunogenicity in nonhuman primates (NHPs), where commercially prepared standard porcine tissue BHVs, implanted in the mitral position, induced chronic Gal-specific stimulation for at least one year.^[26]^ This Gal-specific stimulation was not present in recipient NHPs implanted with tissue valves from genetically modified pigs which do not produce the Gal antigen (GalKO pigs). We have also previously shown in the rat and rabbit subcutaneous implant model that human anti-Gal IgG accelerates calcification of glutaraldehyde-fixed porcine tissue taken from conventional standard pigs, but does not increase calcification of tissues from GalKO pigs.^[25]^ This antibody-induced tissue calcification occurs even after anti-calcification treatment,^[19]^ indicating that this immune mechanism of tissue calcification is independent of passive calcification processes. We^[19,23,25,26]^ and others^[2,20,22,32]^ have indicated that this immune mechanism is a significant cause of tissue calcification which may contribute to SVD, especially in younger patients. BHVs produced from GalKO tissue which does not bind anti-Gal antibody would eliminate anti-Gal antibody induced calcification and improve BHV durability.^[19,20,25]^

To advance towards the use of GalKO porcine tissue in future clinical BHVs, we previously compared the biophysical properties of glutaraldehyde-fixed standard and GalKO pig pericardium.^[24]^ These tissues were shown to be physically equivalent with the same general collagen content and morphology, and showed no significant differences in uniaxial stress characterization or suture retention.^[24]^ To test the biological equivalence of GalKO and standard pig pericardium, we developed a novel surgical valve design which can be produced using either standard or GalKO pig pericardium.^[30]^ In a limited 48-hour duration *in vivo* implantation study using BHVs made from GalKO pig pericardium, we found normal valve function and no acute thrombogenic response.^[30]^ In this study, we extend the biological equivalence testing, comparing mitral valve implantation of GalKO and standard BHVs in the adolescent sheep model for 90 days. To our knowledge this is the first time such a biological equivalence study between GalKO and standard porcine tissue has been reported.

## Materials and Methods

### Porcine Tissue Harvesting and Fixation

The surgical facility’s Animal Care and Use Committee is registered at the CNREEA under the Ethics Committee n°37, Accreditation number: C 75-14-01, and is in compliance with ISO 10993-2 and the Animals (Scientific Procedures) Act of 1986, published by the UK Home Office, and the guidelines for the Care and Use of Laboratory Animals from the US National Institute of Health (Publication No. 85-23, revised 1996). Heart-lung blocks were procured and processed from standard and GalKO pigs in an identical fashion. All tissues were chilled on ice and promptly sent for processing.

Heart-lung blocks were rinsed in saline and the dissected parietal pericardium was cleaned of adherent fat and debris, and carefully examined to discern fiber orientation. The pericardium was placed inside fixation frames and fixed in buffered 0.6% glutaraldehyde (0.6% glutaraldehyde in 20mM HEPES pH 7.4, 26mM MgCl_2_ and 150mM NaCl) for 24 hours at 4 °C. A thickness gauge (Mitutuyo 543-402BS, Sakado, Japan) was used to measure thickness at 12 locations across the tissue. All pericardial sheets were stored in buffered 0.2% glutaraldehyde at 4 °C thereafter.

### Valve Manufacture

**Figure 1:**
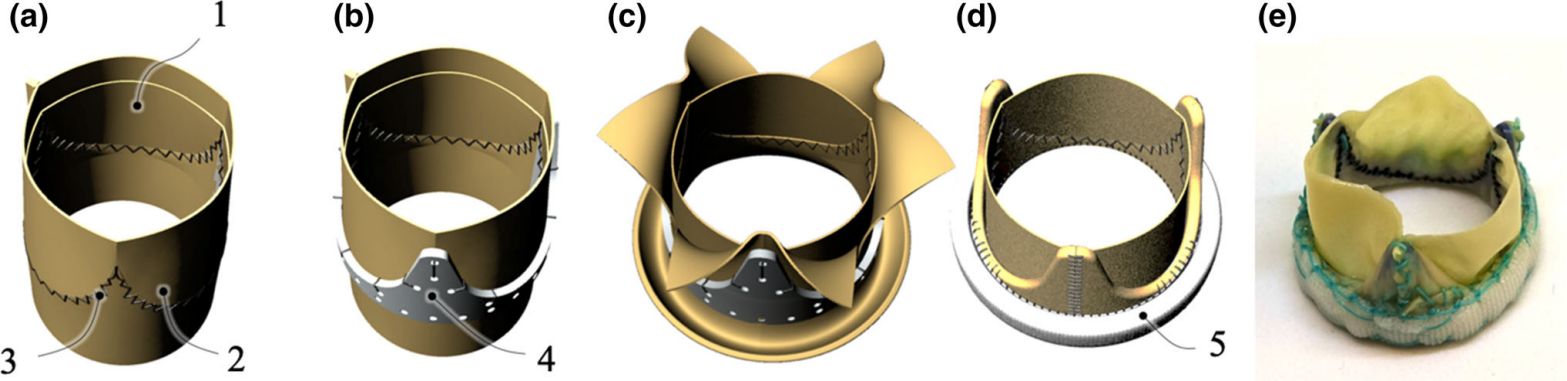
Valve Manufacture. A. Shows the attachment of leaflet-layer pericardial sheet (1) to a wrap-layer pericardial sheet (2) via serrated suturing (3). Subsequently this sheet is sutured into a tube-shape. B. Illustrates the insertion and attachment of pericardial tube into the acetal stent (4). C. and D. Show how the wrap layer is cut and sutured around the stent posts and base. E. A completed valve with attached suture ring (5).

GalKO tissue-type (group 1, *n* = 5) and standard tissue-type (group 2, *n* = 5) BHVs with outside stent diameter 25 mm were constructed, based upon a previously published methodology,^[30]^ as illustrated in Fig. 1. After manufacture, valves were sterilized in buffered 0.6% glutaraldehyde at 20 °C for 2 hours, rinsed in sterile saline, and stored in buffered 0.2% glutaraldehyde at 4 °C until implantation. Valves were quality inspected macroscopically after manufacture and sterilization.

### Animal Implantation and Evaluation

This was a randomized study in which the surgeon and pathologist were blinded to the type of prosthesis. The BHVs were implanted into the mitral position of female adolescent sheep (*Ovis aries*, median age of 13.25 months, range of 11.25–28.75 months at BHV implantation) for 90 days. Mitral valve replacement was carried out under general anesthesia with cardiopulmonary bypass (CPB) and heparinization according standard protocols.^[15]^ Electrocardiography, blood pressure, end-tidal CO_2_, and core body temperature were monitored during the procedure. Epicardial echocardiography was done after the procedure, before chest closure.

Anticoagulation consisted of Enoxaparin (1 mg/kg SC twice a day) and Acetyl Salicylic Acid (250 mg IM once a day) administered for the full 90-day duration of the study, matching the anticoagulation procedures commonly carried out for the first 6–12 weeks after BHV implantation in clinical practice. Transthoracic echocardiography was performed immediately after chest closure and on days 0 + 30, 0 + 60, and 0 + 90. The major echocardiographic parameters assessed were: peak and mean transvalvular pressure drop; prosthetic diameter (both commissure-to-commissure and antero-posterior); mitral valve perimeter; valvular regurgitation; and leaflet motion. Blood samples were collected for biochemistry, hemolysis, complete blood count and coagulation parameter analyses at baseline, Day 0 + 30, Day 0 + 60 and Day 0 + 90.

### Elective Termination Studies

After 90 days, echocardiography and left ventriculography were carried out on each surviving animal. Each sheep was then heparinized, humanely euthanized and a complete necropsy performed. The heart and lungs were excised and separated, followed by incision of the heart to expose the BHV and enable thrombus inspection of the cardiac chambers.

Each BHV was macroscopically evaluated for the presence of pannus, vegetation, inflammation, thrombus, mineral deposits, and visible SVD. These levels of pannus, thrombus, and calcification were graded according to previously established criteria^[4,33]^ (Supplemental Data Table S1). Mineral deposit analysis and any stent fracture of the BHV was also assessed by x-ray (Faxitron® MX-20 Cabinet X-Ray system, Wheeling, Illinois). The lungs, brain, kidneys and liver were macroscopically and microscopically examined, documenting any infarcts, lesions, or other abnormalities. The tissue cusps were removed from each BHV and split down the middle into two pieces. One half was used for microscopic histological evaluation, and the other half analyzed by quantitative atomic spectroscopy (QAS) to quantify calcium levels.

#### Histology

Explanted BHV leaflet samples were fixed in 4% neutral buffered formalin for 48 hours, embedded in paraffin and 5 µm sections were stained with Hematoxylin-Eosin & Saffron (HE&S), Movat Pentachrome, Masson Trichrome (for connective tissue), Azan Mallory (for fibrin), Red Alizarin (for calcium) and Gram stain (for bacteria).

#### Quantitative Atomic Spectroscopy

Elemental analysis of calcium and phosphorus content was undertaken by inductively coupled plasma atomic emission spectroscopy (ICP-AES iCAP 7400, Thermo, France). Calcium content is reported as µg/mg of dried tissue.

### Statistics

Continuous variables are expressed as mean ± standard deviation. Primary endpoints for the study—cardiac thrombus deposition, pannus formation, inflammation and vegetation, calcification, visible SVD, and tissue histopathology—were assessed by Mann-Whitney U tests. Secondary endpoints of haemodynamic valve function, in terms of leaflet motion, regurgitation, and transvalvular pressure drop, were also examined via Mann-Whitney U tests, whilst mortality comparison, which is binary, was analyzed using a Fisher’s exact test. A *p*-value < .05 is regarded as significant for analyses comparing end points between GalKO and standard tissue valves. All analyses were performed using the SPSS 26.0.0.0 software package (SPSS, Chicago, Illinois).

## Results

### Recipient Survival and Health

Nine of 10 BHV recipients (group 1 [GalKO tissue-type], *n* = 5; and group 2 [standard tissue-type], *n* = 4) were healthy throughout the study and completed the 90-day postoperative period. The median age of the sheep at implant were 13.5 and 13.1 months for group 1 and group 2 respectively. The mean weight of the sheep at implant were 55.8 and 58.5 kg for group 1 and group 2 respectively. Full details of sheep age and weight at implant can be found in the Supplemental Data. One animal who had received a standard pericardial valve (group 2) died from the well-recognized non-valve-related bloating syndrome on day 0+53. Haemodynamics measured from echocardiography on day 0+30 for this recipient showed normal valve function, with a mean transvalvular pressure drop of 2.5 mmHg and trivial valvular regurgitation. At necropsy there were no paravalvular defects or leaflet lesions, with pliant cusps, and no cardiac abnormalities.

There were no adverse valve-related events during this study. All 9 surviving animals demonstrated excellent haemodynamic performance throughout the study. Recipient sheep showed no clinically relevant abnormal haemotology, blood chemistry, or hemolysis levels, with no differences between the two study groups. Plasma free hemoglobin values, 0.0074 g/dL ± 0.0028 and 0.0098 g/dL ± 0.0056 for groups 1 and 2 respectively, indicated that neither group induced excessive hemolysis. Full blood biochemistry, hemolysis, coagulation, and hematology measurements can be found in the Supplemental Data. At autopsy, no valve related differences were detected between the explanted recipient organs.

### Echocardiography

**Figure 2:**
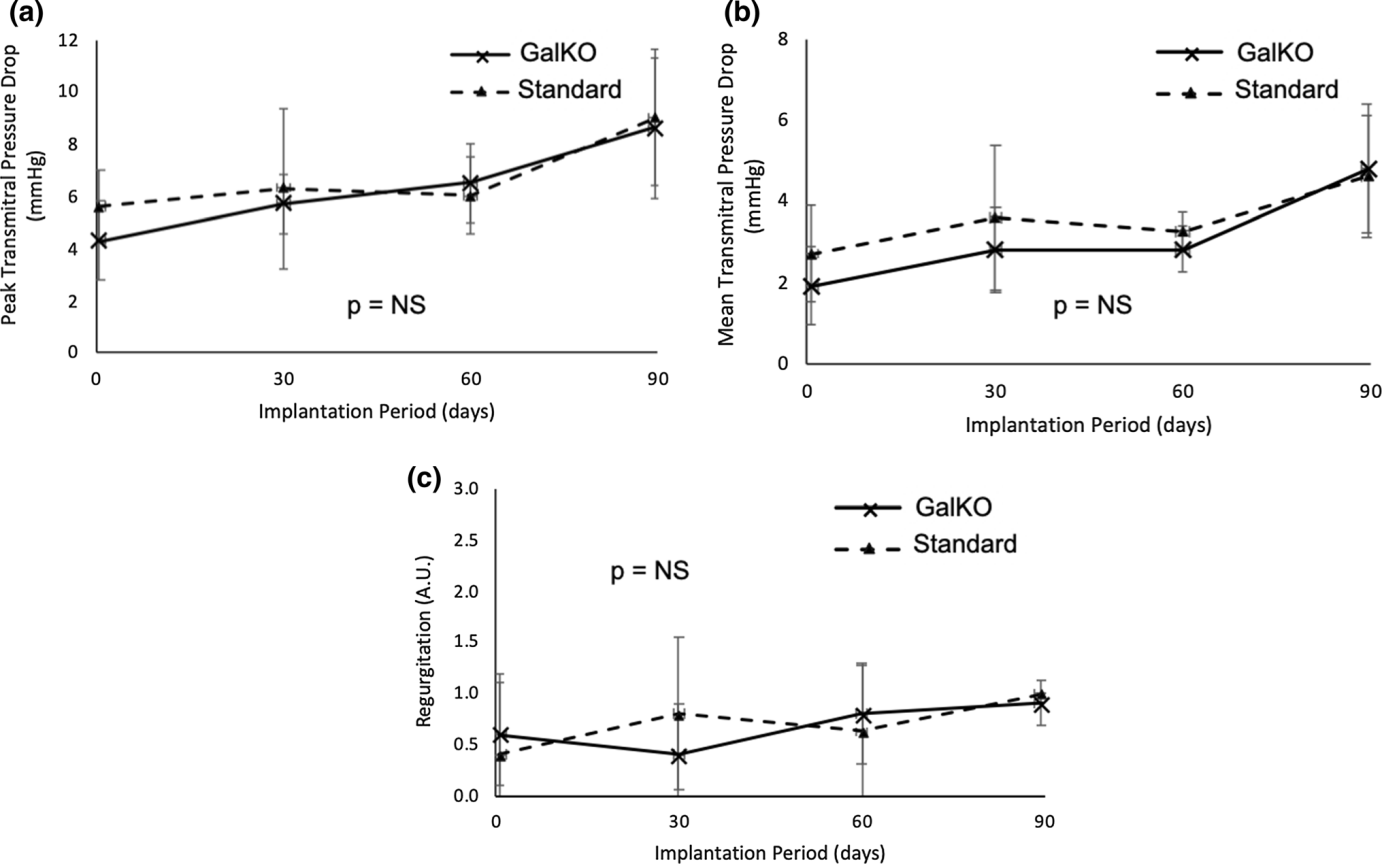
Valve haemodynamics. (**a**) Peak transmitral pressure drop. (**b**) Mean transmitral pressure drop. (**c**) Mitral regurgitation. All values given are for the mean ± standard deviation.

There was no difference between study groups for echocardiographic hydrodynamic data taken on days 0, 0 + 30, 0 + 60, and 0 + 90 of the study. Peak and mean transmitral pressure drops were stable for both study groups, ranging between 4 and 6 mmHg (peak pressure) and 2-4 mmHg (mean pressure) for the majority of the study (Figs. 2a and 2b). There was a small increase in the mean and peak transmitral pressure drops on day 0+90 for both groups. There was no difference in peak or mean pressure drops between the two groups (*p* = 0.89 for peak pressure drop and *p *= 0.39 for mean pressure drop, group 1 versus group 2). Measurements of prosthetic diameters, both commissure-to-commissure and antero-posterior, were between 2 and 3 cm for all BHVs throughout the study, and the mitral valve perimeter remained between 6 and 7 cm, with no difference between study groups. Mitral valve regurgitation was minimal and remained stable for both groups during the study (Fig. 2C). No reduction in leaflet motion or coaptation was observed for any valves in either group. Left ventriculography on day 0+90 showed only trivial regurgitation in both groups.

### Valve Morphology at Explant

**Figure 3:**
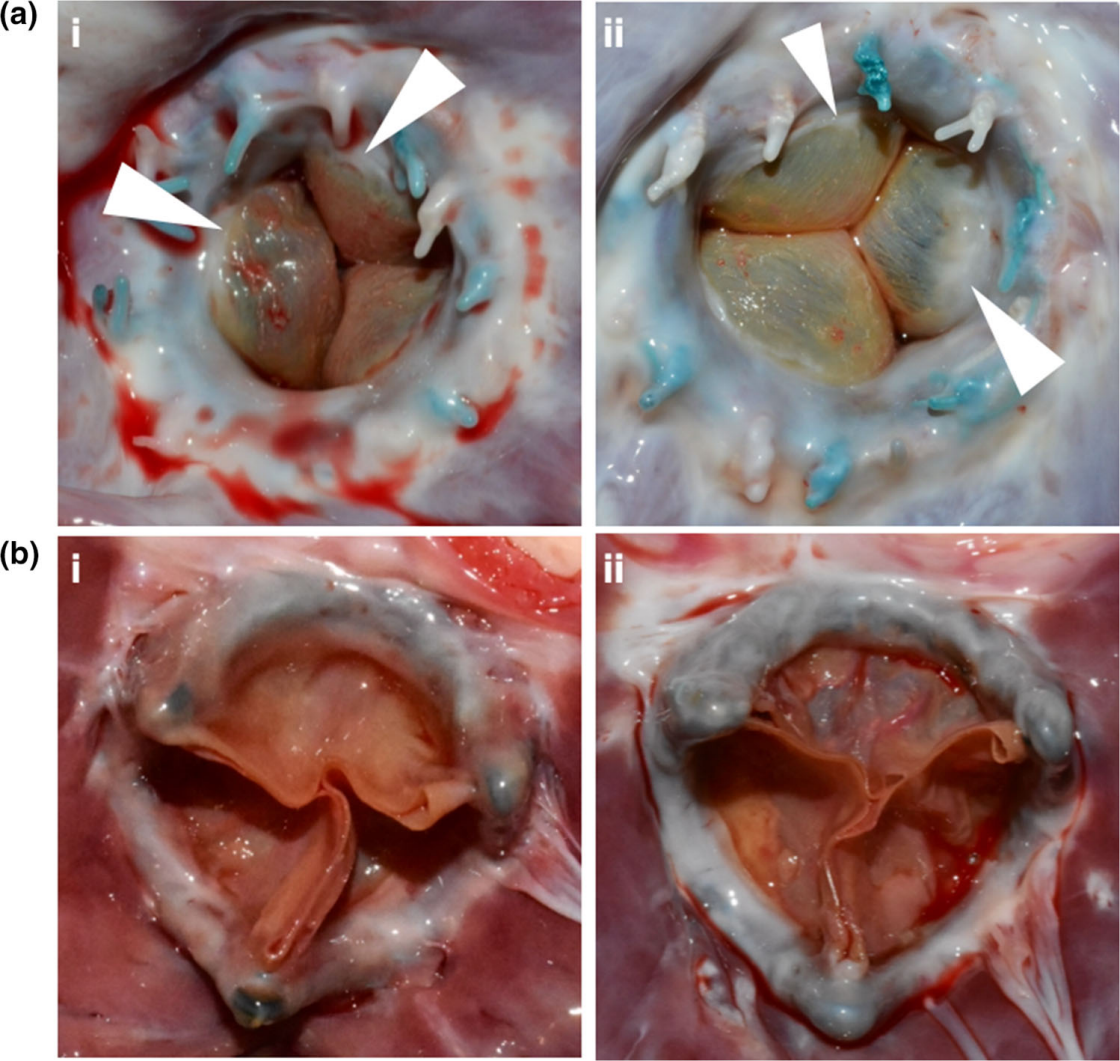
Explanted prosthetic valves. (**a**) Inflow view of (i) GalKO valve and (ii) Standard valve. (**b**) Outflow view of (i) GalKO valve and (ii) Standard valve. Arrows indicate sites of pannus formation on the leaflets.

Inflow and outflow views of representative explanted BHVs from both study groups are presented in Fig. 3. There was good integration with the host tissue for all BHVs. Mean grades for macroscopic observations of pannus, thrombus, and calcification formation are presented in Table 1. Pannus formation, most prominent on the pericardium-covered stent with occasional minimal coverage of the leaflets at the insertion end (Fig. 3A) was graded as 1.0 for all BHVs. Minimal thrombus deposition was evident in most valves, scoring 1.0, with no observed difference in severity or location between the two study groups. All BHV leaflets appeared pliant and mobile with no stiff deposits, graded 0.8 ± 0.24 for BHVs from group 1 and 0.8 ± 0.43 for BHVs from group 2. No signs of SVD, such as stent fracture or leaflet tears, were detected.

**Table 1:** Macroscopic evaluation.

Pathology	Group			
	Group 1 (*n* = 5)		Group 2 (*n* = 4)	
	Mean	SD	Mean	SD
Pannus	1.00	–	1.00	–
Thrombus	1.00	–	1.00	–
Calcification	0.80	0.24	0.80	0.43

**Figure 4:**
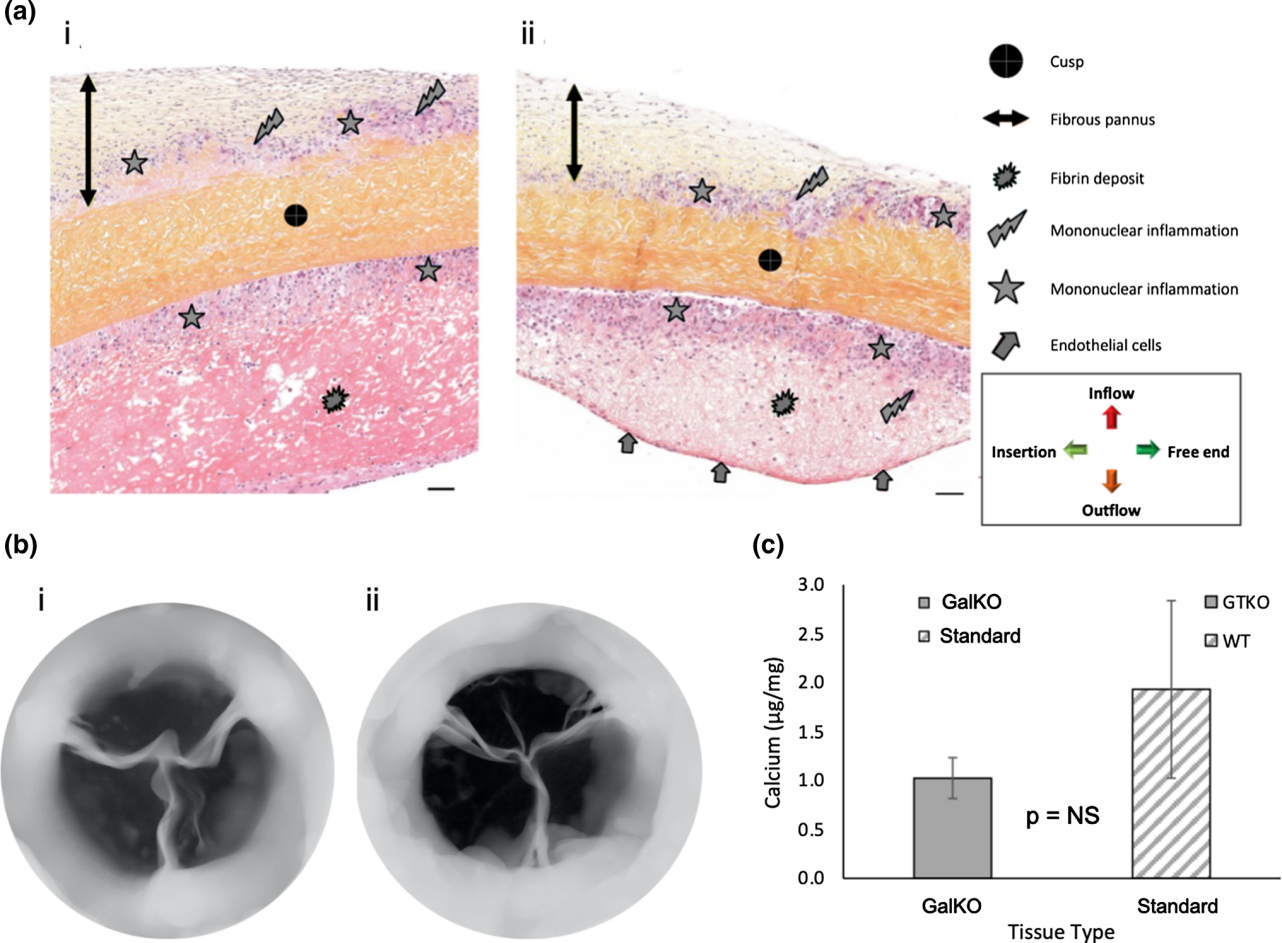
Microscopic analysis. (A) Bioprosthetic cusp HE&S histology. i. Medial cusp GalKO valve, explanted on Day 0+91. ii. Lateral cusp from a standard valve, explanted on Day 0+91. Scale bar 50 µm in all images. (B) Inflow x-ray calcification analysis views of (i) a representative GalKO valve; (ii) a representative standard valve. C. Calcium levels determined by quantitative atomic spectroscopy for the two tissue types.

There were no notable differences in leaflet histology between the study groups. A mild level of fibrin deposition, pannus formation and inflammatory cellular infiltrate (mainly macrophages and lymphocytes with minimal foreign body cells) was present in most sections from both groups (Fig. 4A, Supplemental Figs. S1 and S2, Supplemental Table S2). In some leaflets there was evidence of endothelial cell colonization on the surface of the pannus. This tended to occur more frequently with standard porcine pericardial leaflets. Gram staining detected no evidence of bacterial growth.

### Tissue Calcification

Red Alizarin stains found no evidence of tissue calcification in the valve leaflets. Consistent with this, X-ray examination revealed no or minimal radio-dense areas characteristic of mineral deposits (Fig. 4B). Focal radio-dense areas were mainly distributed on the sewing ring or around the base and commissures of the leaflets, and were pin-point lesions no larger than 1 mm. The levels of calcium detected by QAS (Fig. 4C) were 1.0 µg/mg ± 0.2 for GalKO BHVs (*n* = 3) and 1.9 µg/mg ± 0.9 for standard BHVs (*n* = 3), and were considered low and equivalent (*p* = 0.40).

## Discussion

In this study, the standard mitral valve implantation model in adolescent sheep, with 90-day follow up, was used to compare the biological equivalence of genetically engineered GalKO pericardium and standard pig pericardium. We found that BHVs made with either tissue type showed equivalent performance. There were no valve related deaths and there were no GalKO tissue related effects on recipient hematology, blood chemistry, coagulation or hemolysis. The haemodynamic performance of the BHVs throughout the study period was similar for both tissue types (Figs. 2A–2C), within the range of *in vitro* data previously determined for this valve design.^[30]^ At explant, both types of BHVs demonstrated good local integration with host tissue (Fig. 3), with no signs of excessive inflammation or vegetation (Fig. 4A). Mild pannus development, mainly around the circumference of the valve ring, was observed equally in all explanted BHVs. The minimal levels of fibrin deposition observed on the leaflet surface and the minimal plasma free hemoglobin values indicate that both study groups present an acceptable and equal level of thrombogenicity, with no valve specific hemolysis. No valve in either study group showed evidence of SVD. In this study the degree of tissue calcification, measured by x-ray, Red Alizarin histology or QAS, was low and considered equivalent in both tissue types. This low level of tissue calcification is comparable to similar analyses from other published studies (Supplemental Data Table S3).^[7,10,11,15,34]^

Valves made from either tissue had low peak and mean transvalvular pressure drops for the majority of the implantation period but showed a moderate increase in transmitral pressure drop after day 0 + 60 (Figs. 2a and 2b). This likely occurred due to evolving prosthetic size mismatch as a result of animal growth, with an associated transvalvular pressure loss for the now undersized valve. Taking this consideration into account, haemodynamic measurements taken throughout the study were consistent between the groups and showed good performance over the 90-day implantation period. The absence of any difference in clinical, functional, pathological or mineralization criteria between the two tissue types indicates biological equivalence between GalKO and standard porcine pericardium. Accordingly, this suggests for the first time that GalKO tissue can be a suitable replacement for standard porcine tissue in the construction of surgical or transcatheter BHVs.

Commercial surgical BHVs use either glutaraldehyde-fixed porcine valves or bovine pericardium, while porcine or bovine pericardium is employed in transcatheter valve replacement (TVR).^[5]^ Porcine pericardium is advantageous in TVR, because it is 30-40% thinner than bovine pericardium. This enables TVR devices incorporating porcine pericardial leaflets to be crimped into a smaller diameter catheter, reducing the risk of vascular complications during device delivery. Porcine pericardial leaflets could also be beneficial in surgical BHVs, as the thinner leaflet material is more flexible and increases the effective orifice area of the valve. The long-term durability of TVR remains to be determined, however the comparable short- and mid-term outcomes for porcine and bovine pericardial TVR,^[5]^ and the performance of the surgical porcine pericardial BHVs in this study and ongoing durability studies^[30]^ suggest that the potential of surgical porcine pericardial heart valves should continue to be investigated.

There is evidence that the daily clinical immune discordance of Gal antigen on current commercial BHVs and anti-Gal antibody in patients contributes to clinical BHV calcification and increases the occurrence of SVD,^[25]^ especially in younger patients. This original study of biological equivalence between GalKO and standard pig pericardium is an essential step towards the future clinical use of BHVs produced from porcine GalKO tissue. It is worth noting that in this study, using standard adolescent sheep, there is no circulating anti-Gal antibody, so the degree of tissue calcification was expected to be the same in both groups, as observed. Further, the age of animals in this study may limit the degree of tissue calcification, however this equivalence study demonstrating comparable levels of calcification between standard pig pericardium and GalKO pericardium is relevant. Future studies using a host species which expresses anti-Gal antibody would be an important step in understanding the putative anti-calcification advantage afforded by BHVs constructed with GalKO tissue. We also note that in anticipation of future changes in BHV testing standards, recent studies have investigated BHV performance in sheep with a 140–150 day study period. However, the 90-day follow up duration in this study is still consistent with current 5840-2 International Standards requirements,^[3]^ is consistent with previous studies^[11,15]^ and is sufficient to detect serious valve dependent pathologies which would prevent use of GalKO tissue. Additionally, individual studies have noted that extending BHV implantation in sheep from 3 months to 6 months does not increase calcification further.^[11]^ Likewise the International Standards do not specify the number of animals used for *in vivo* studies,^[3]^ but previous studies have used similar or fewer animals for equivalent *in vivo* BHV comparisons.^[11,15,27]^ We also acknowledge that as no significant changes were observed during this 90-day period, a follow-up study over 150-days would be beneficial before clinical application. The impact of withdrawal of anticoagulants could also be studied.

## Conclusions

Surgical BHVs were constructed using genetically engineered GalKO pericardium and standard pig pericardium and tested in the standard mitral valve replacement model in adolescent sheep. Tissue equivalence was evaluated based on clinical, functional, pathological and mineralization criteria. There was no difference in these parameters at 90 days between the two tissue types, indicating biological equivalence of GalKO and standard porcine pericardium. These results indicate that GalKO pig pericardium is suitable for use in artificial heart valves, and suggest that commercial adoption of GalKO tissue for surgical or transcatheter BHVs will eliminate the current clinical anti-Gal antibody incompatibility to improve BHV resistance to tissue calcification and SVD. The commercial cost of using genetically modified tissue for BHV production is an important consideration. We believe the high cost of current commercial BHVs, with the pig cost being a relatively small part, leaves room for what we calculate, albeit imperfectly, would be an acceptable premium for a top-quality product. Initial cost mitigation from reduced surgical reoperations in the event of extended prosthesis functionality is also an important consideration.

## Supplementary Information

Below is the link to the electronic supplementary material.

Supplementary file1 (XLSX 42 kb)

Supplementary file2 (DOCX 1501 kb)

## Abbreviations

BHV: Biological heart valve
CPB: Cardiopulmonary bypass
Gal: Galactose-alpha-1,3-galactose
GalKO: GGTA-1 Glycosyltransferase knockout
HE&S: Hematoxylin-Eosin & Saffron
MHV: Mechanical heart valve
NHP: Non-human primate
QAS: Quantitative atomic spectroscopy
SVD: Structural valve degeneration
TVR: Transcatheter valve replacement
