# Supplementary material for: Biological Equivalence of GGTA-1 Glycosyltransferase Knockout and Standard Porcine Pericardial Tissue Using 90-Day Mitral Valve Implantation in Adolescent Sheep
Source: Cardiovasc Eng Technol. 2021 Nov 24;13(3):363–72. doi: 10.1007/s13239-021-00585-0 (PMC9197892; doi:10.1007/s13239-021-00585-0)
Supplement: Supplementary file 2 — Supplementary file2 (DOCX 1501 kb) [file 13239_2021_585_MOESM2_ESM.docx]

# Supplementary Data for ‘Biological Equivalence of GGTA-1 Glycosyltransferase Knockout and Standard Porcine Pericardial Tissue using 90-day Mitral Valve Implantation in Juvenile Sheep’

The supplementary data provides additional information on: Ovine serum biochemistry, coagulation, haemolysis and haematology results for GalKO and Standard BHV recipients at 0, 30, 60 and 90 days post mitral valve implantation (Excel file: Supplemental Serum and Haematology Results); Details of the macroscopic evaluation grading scheme (Table S1); Additional histology results examining connective tissue integrity (Figure S1) and pannus and fibrin deposition (Figure S2) are provided as is a summary of histological features (Table S2). We also provide a summary of tissue calcification results from comparable *in vivo* studies (Table S3).

## Ovine Biochemistry

Individual and average group results for ovine hematology and biochemistry studies are presented in the Supplemental Serum and Haematology Results. Parameters presented are: **Biochemistry parameters**: Glucose, Creatinine, Urea, Calcium, Chloride, Sodium, Potassium, Bicarbonate, Phosphorus, Plasma Proteins, Magnesium, Total Bilirubin, Gamma Glutamyl Transferases, Aspartate Aminotransferase (AST), Alanine Aminotransferase (ALT) and Alkaline Phosphatase; **Haemolysis parameters:** Plasma Free Haemoglobin; **Haemostasis parameters:** Prothrombin ratio (PR), Activated Partial Thromboplastin Time (aPTT) and Fibrinogen; **Complete Blood Count** (CBC): Red Blood Cells (RBC), Haemoglobin, Haematocrit, Mean Corpuscular Volume (MCV), Mean Corpuscular Haemoglobin Concentration (MCHC), Mean corpuscular Haemoglobin (MCH), White Blood Cells (WBC), Platelets, and Reticulocytes.

## Macroscopic evaluation grading

Table S1: Macroscopic observations of explanted valves were graded from 0 - 4 according to previously established criteria [1,2] to enable semi-quantification of pannus, thrombus, and calcification levels from macroscopic observations. Mean values and standard deviation for each group for each parameter are presented in Table 1 of the manuscript.

Table S1 - Macroscopic evaluation grading

| Grade | Pannus* | Thrombus | Calcification** |
| --- | --- | --- | --- |
| 0 | No pannus | Absent | Absent |
| 1 | Mild and involving part of the circumference of the valve ring | Minimal,  1-25% of leaflet covered | Focal (pin-point),  < 1 mm diameter |
| 2 | Moderate and extending up to 2 mm onto the surface of the cusp | Moderate,  26-50% of leaflet covered | Focal,  > 1 mm diameter or multiple |
| 3 | Severe and extending beyond 2 mm onto the surface of the cusp | Severe,  51-75% of leaflet covered | Multiple,  > 1 mm diameter |
| 4 | Very severe and surrounding the flow and non-flow surfaces of the cusp, causing the cusp to shorten | Extensive,  76-100% of leaflet covered | Massive deposition |

*Pannus classification derived from Butany et al. [1]; **Calcification classification derived from Valente et al. [2].

## Histology

Three sections perpendicular to the free edge of the cusps were performed from each of the bioprosthetic cusps from 3 valves from each group, and embedded in paraffin. Five μm thick paraffin cusp sections were stained with Hematoxylin-Eosin & Saffron (HE&S), Movat Pentachrome, Masson Trichrome, Azan Mallory, Red Alizarin, and Gram stain.

Table S2 summarises the histology results. There was minimal dissociation of collagen fibres or eosinophilic fluid infiltration in explanted BHV leaflets from the two groups based on uniform Masson’s trichrome staining (Figure S1). Mild fibrous pannus and fibrin deposits were commonly seen in both groups (Figure S2). The collated findings for the histology are presented in Table S2. This analysis found minimal dissociation of collagen fibres. Mild fibrin and pannus deposits commonly seen in both study groups were present on both sides of the cusp but more commonly detected on the inflow surface. Mild reendothelialization was observed in both groups, usually located on the fibrous pannus surface, the fibrin deposits and occasionally on the cusp surface devoid of pannus, but was more common in group 2 than in group 1 (19/27 vs 9/27 respectively). Almost all examined sections contained mononuclear inflammatory cells, primarily macrophages, between the pannus and leaflet tissue and occasionally lymphocytes within the fibrous pannus, with little difference in occurrence between the two groups. Multinucleated giant cells were also observed at the interface between pannus and leaflet tissue in some of the sections from both groups, with the prevalence very similar over the two groups.

Table S2 - Summary of microscopic findings from leaflet histology

| Group | Group 1 (N = 3, 27 sections) | | | Group 2 (N = 3, 27 sections) | | |
| --- | --- | --- | --- | --- | --- | --- |
| Diagnosis | Incidence | Mean severity | SD | Incidence | Mean severity | SD |
| Dissociation of collagen fibres | 2/27 | 1.00 | 0.00 | 2/27 | 1.00 | 0.00 |
| Fibrin deposits | 25/27 | 1.56 | 0.65 | 26/27 | 1.31 | 0.62 |
| Fibrous pannus | 21/27 | 1.24 | 0.44 | 22/27 | 1.77 | 0.69 |
| Reendothelialization | 8/27 | 1.88 | 0.83 | 19/27 | 1.63 | 0.68 |
| Mononuclear inflammatory cells | 26/27 | 1.50 | 0.65 | 24/27 | 1.38 | 0.58 |
| Multinucleated giant cells | 12/27 | 1.08 | 0.29 | 11/27 | 1.00 | 0.00 |

Group incidence – number of sections with finding; Mean severity – sum of severity scores of all sections with finding combined, divided by number of sections with finding; SD – standard deviation; Grading – 0 – none, 1 – minimal; 2 – mild, 3 – moderate, 4 – marked, 5 - severe. Dissociation of collagen fibres – separation of collagen fibres from each other in the leaflet, either due to fibrin deposits or infusion of eosinophilic fluid (plasmatic insudation); Fibrin deposits – presence of non-globular protein deposits, associated with thrombin and blood clotting, on the cusp surface; Fibrous pannus – the presence of a layer of fibrovascular tissue growth over the cusp surface; Reendothelialization – the growth of barrier layers between blood vessels and the leaflet surface tissue; Mononuclear inflammatory cells – presence of lymphocytes, macrophages and plasma cells at the interface between any pannus and the leaflet tissue; Multinucleated giant cells – presence of masses formed from several distinct cells at the interface between any pannus and the leaflet tissue.

Figure S1


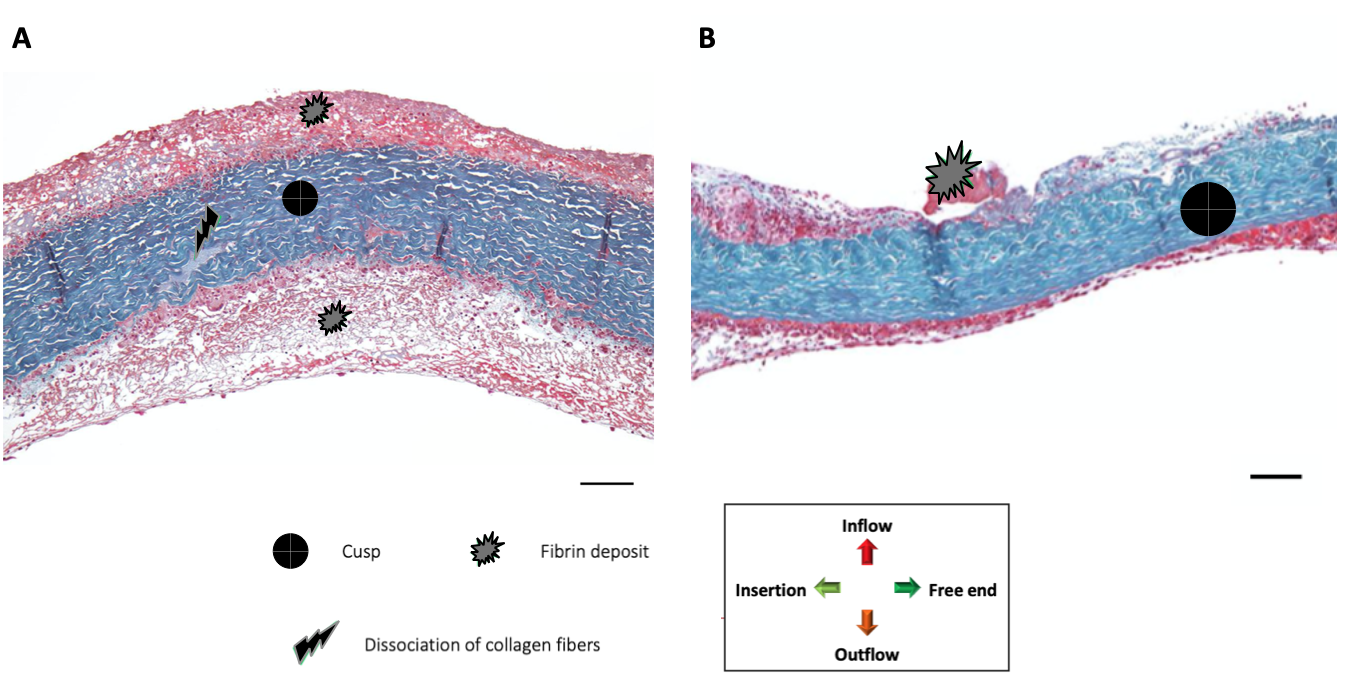


Figure S1: Bioprosthetic cusp connective tissue histology. A. GalKO BHV cusp stained with Masson’s trichrome explanted on day 0+89. B. Standard porcine BHV cusp stained with Masson’s trichrome explanted on day 0+91. Scale bar 100 µm in all images.

Figure S2


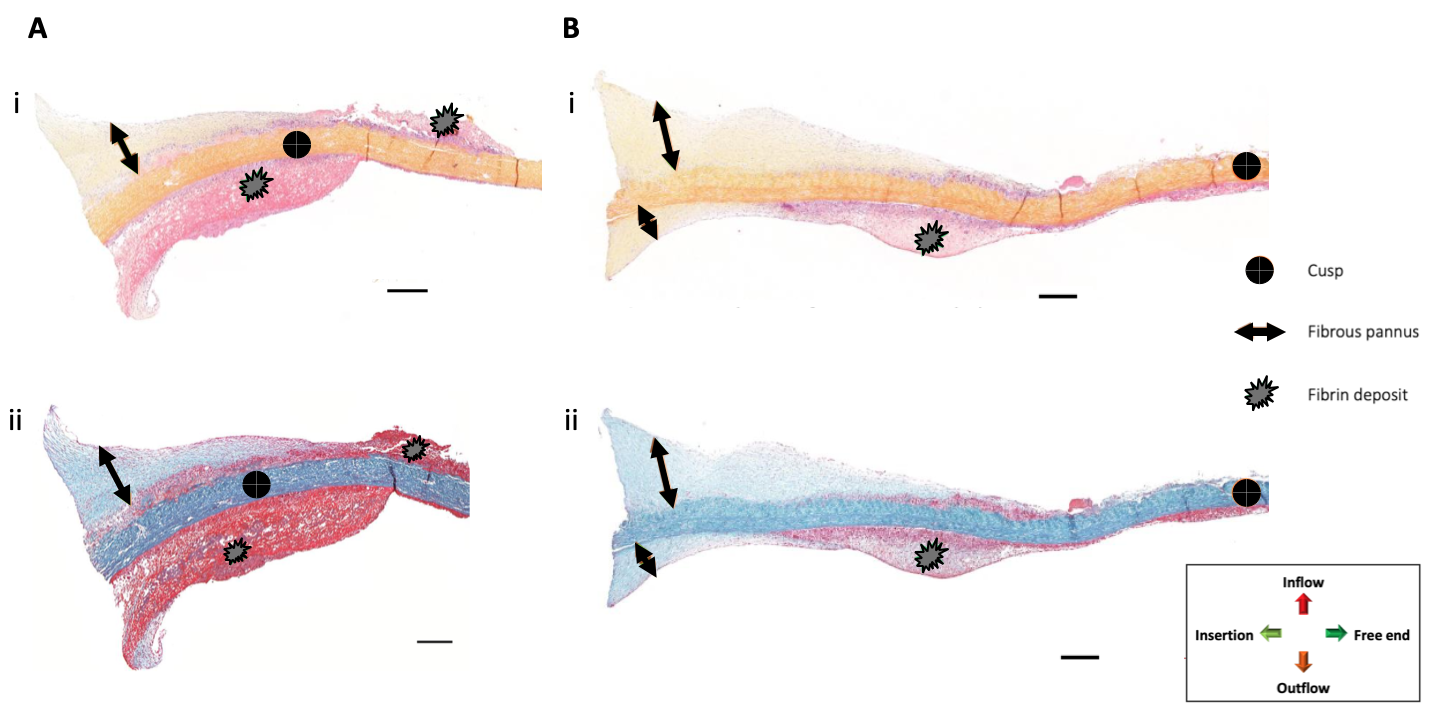


Figure S2: Bioprosthetic cusp fibrous pannus and fibrin deposit histology. A GalKO medial BHV cusp stained with. i. HE&S and ii. Azan Mallory at explanted on day 89. B. Standard porcine lateral BHV cusp stained with i. HE&S and ii. Azan Mallory at explanted after 91 days. Scale bar 250 µm in all images.

## Previous Tissue Calcification Studies

In our study the level of tissue calcium detected by QAS (Figure 5B) was 1.0 µg/mg ± 0.2 for GalKO BHVs (n=3) and 1.9 µg/mg ± 0.9 for standard BHVs (n=3). There was no evidence of tissue calcification based on Red Alizarin histology and there was minimal radio dense regions of BHV x-rays. While no two studies are directly comparable these results are broadly similar to a variety of sheep implant studies listed in Table S3 which reported low levels of tissue calcification after 90 and 150-day of implantation for a wide range of bovine and porcine BHVs.

Table S3: Tissue calcification results from previous studies

| Study | Tissue | Study period | Assessment method | Calcium level | Study comments |
| --- | --- | --- | --- | --- | --- |
| Harvey et al. 2015 [3] | Bovine pericardial valve | 90 days | Macroscopic | No signs | Low/no level of calcification |
| Connolly et al. 2011 [4] | Porcine aortic valve | 150 days | Macroscopic | No gross calcification | Low calcification |
|  |  |  | QAS | 6.0 µg/mg |  |
| Flameng et al. 2006 [5] | Bovine pericardial and porcine aortic valves | 90 days | QAS | 14.3 µg/mg | Average calcification level across range of valves |
| Flameng et al. 2008 [6] | Bovine pericardial valve | 150 days | Histology | Trace | Low levels of calcification |
|  |  |  | QAS | 1.1 µg/mg |  |
| Weber et al. 2006 [7] | Porcine aortic valve | 144 days | Histology | None observed | Low/no level of calcification |
|  |  |  | QAS | 1.97 µg/mg |  |
|  |  |  | X-ray | 1.15 |  |

All data taken from studies of tissue bioprostheses implanted in mitral position of juvenile sheep. QAS – Quantitative Atomic Spectroscopy.

## Supplementary Data References

[1] J. Butany, V. Nair, S.W. Leong, G.S. Soor, C. Feindel, Carpentier-Edwards Perimount valves - Morphological findings in surgical explants, J. Card. Surg. 22 (2007) 7–12.

[2] M. Valente, F. Laborde, G. Thiene, P. Gallix, F. Calabrese, E. Talenti, U. Bortolotti, A. Piwnica, EvaIuation of Pericarbon vaIve prosthesis : In vitro, ultrastructural, and animal studies, J. Card. Surg. (1989) 79–88.

[3] L. Harvey, R. Bianco, M. Lahti, J. Carney, L. Zhang, N. Robinson, Carpentier-Edwards aortic pericardial bioprosthetic valve as a valid control in preclinical in vivo ovine studies, Eur. J. Pharmacol. 759 (2015) 192–199.

[4] J.M. Connolly, M.A. Bakay, I.S. Alferiev, R.C. Gorman, J.H. Gorman, H.S. Kruth, P.E. Ashworth, J.K. Kutty, F.J. Schoen, R.W. Bianco, R.J. Levy, Triglycidyl amine crosslinking combined with ethanol inhibits bioprosthetic heart valve calcification, Ann. Thorac. Surg. 92 (2011) 858–865.

[5] W. Flameng, B. Meuris, J. Yperman, G. De Visscher, P. Herijgers, E. Verbeken, Factors influencing calcification of cardiac bioprostheses in adolescent sheep, J. Thorac. Cardiovasc. Surg. 132 (2006) 89–98.

[6] W. Flameng, B. Meuris, G. De Visscher, C. Cunanan, E. Lane, E. Verbeken, P. Herijgers, M.C. Herregods, Trilogy Pericardial Valve: Hemodynamic Performance and Calcification in Adolescent Sheep, Ann. Thorac. Surg. 85 (2008) 587–592.

[7] P.A. Weber, J. Jouan, A. Matsunaga, E. Pettenazzo, T. Joudinaud, G. Thiene, C.M.G. Duran, Evidence of mitigated calcification of the Mosaic versus Hancock Standard valve xenograft in the mitral position of young sheep, J. Thorac. Cardiovasc. Surg. 132 (2006) 1137–1143.
